# Microneedle-Mediated Treatment of Obesity

**DOI:** 10.3390/pharmaceutics17020248

**Published:** 2025-02-13

**Authors:** Huanhuan Pan, Wanshan Hu, Chunxian Zhou, Jubo Jian, Jing Xu, Chao Lu, Guilan Quan, Chuanbin Wu, Xin Pan, Tingting Peng

**Affiliations:** 1 State Key Laboratory of Bioactive Molecules and Druggability Assessment, Guangdong Basic Research Center of Excellence for Natural Bioactive Molecules and Discovery of Innovative Drugs, College of Pharmacy, Jinan University, Guangzhou 511436, China; panhuan@stu2024.jnu.edu.cn (H.P.); huwanshan3@stu2020.jnu.edu.cn (W.H.); pk0818@stu.jnu.edu.cn (C.Z.); jian552@stu.jnu.edu.cn (J.J.); 2023102196xj@stu2023.jnu.edu.cn (J.X.); chaolu@jnu.edu.cn (C.L.); quanguilan@jnu.edu.cn (G.Q.); chuanbinwu@jnu.edu.cn (C.W.); 2 Jiangmen Wuyi Hospital of Traditional Chinese Medicine, Affiliated Jiangmen Traditional Chinese Medicine Hospital of Jinan University, Jiangmen 529031, China; 3 School of Pharmaceutical Sciences, Sun Yat-sen University, Guangzhou 510006, China

**Keywords:** microneedle, drug penetration, obesity, treatment, prospect

## Abstract

Obesity has become a major public health threat, as it can cause various complications such as diabetes, cardiovascular disease, sleep apnea, cancer, and osteoarthritis. The primary anti-obesity therapies include dietary control, physical exercise, surgical interventions, and drug therapy; however, these treatments often have poor therapeutic efficacy, significant side effects, and unavoidable weight rebound. As a revolutionized transdermal drug delivery system, microneedles (MNs) have been increasingly used to deliver anti-obesity therapeutics to subcutaneous adipose tissue or targeted absorption sites, significantly enhancing anti-obese effects. Nevertheless, there is still a lack of a review to comprehensively summarize the latest progress of MN-mediated treatment of obesity. This review provides an overview of the application of MN technology in obesity, focusing on the delivery of various therapeutics to promote the browning of white adipose tissue (WAT), suppress adipogenesis, and improve metabolic function. In addition, this review presents detailed examples of the integration of MN technology with iontophoresis (INT) or photothermal therapy (PTT) to promote drug penetration into deeper dermis and exert synergistic anti-obese effects. Furthermore, the challenges and prospects of MN technology used for obesity treatment are also discussed, which helps to guide the design and optimization of MNs. Overall, this review provides insight into the development and clinical translation of MN technology for the treatment of obesity.

## 1. Introduction

Obesity represents a critical public health challenge in the 21st century, affecting over 2 billion adults globally who are classified as overweight or obese. By 2035, the global population of overweight or obese individuals is estimated to exceed 4 billion, resulting in a dramatic increase in the global prevalence from 38% to over 50% [1]. Additionally, the prevalence of overweight and obesity among children and adolescents has increased sharply in the past decade [2,3]. In 2013, obesity was classified as a chronic disease by the American Medical Association, showing a close relationship with a variety of diseases such as metabolic disorder, cardiovascular disease, hypertension, and cancers [4,5]. Obesity poses a huge burden to the healthcare system and society. Current therapeutic approaches for obesity include dietary management, physical exercise, surgical interventions, and anti-obesity medications. Although lifestyle interventions are fundamental to obesity treatment, patients often find it difficult to adhere to long-term self-discipline, leading to weight loss failure. Even when weight loss is achieved through diet and exercise, it is often followed by rebound weight gain. Moreover, for severely obese patients, reliance on dietary and exercise control alone may be insufficient to achieve optimal weight loss. Surgery, such as liposuction, provides an effective means of removing subcutaneous fat but is highly invasive and poses risks, including infection, scarring, hematoma, deep vein thrombosis, pulmonary embolism, and anesthesia-related complications [6,7]. Anti-obesity drugs mainly act by targeting the central nervous system to suppress appetite or the gastrointestinal system to hinder absorption [8], typically administered orally or by injection. However, subcutaneous injections of lipolysis-promoting drugs may lead to local burning sensations, erythema, and granulomatous reactions, including infections [9,10]. Oral anti-obesity drugs may cause severe adverse effects such as gastrointestinal issues, cardiovascular toxicity, headaches, and insomnia [11]. Currently, non-invasive techniques like laser therapy, high-intensity focused ultrasound, cryolipolysis, and radiofrequency are being investigated for localized fat reduction [12,13,14]. However, these methods require specialized equipment and must be conducted by trained clinicians. Consequently, the development of innovative, effective, and safe treatment strategies for obesity has become an urgent priority.

The hallmark characteristic of obesity is the excessive accumulation of subcutaneous fat. Adipose tissue, a specialized connective tissue predominantly composed of adipocytes, can be classified into two types: white adipose tissue (WAT) and brown adipose tissue (BAT), which differ in structure, location, and function. The excessive accumulation and hypertrophy of adipocytes in WAT are key pathological features of obesity. White adipocytes have fewer mitochondria in their cytoplasm, whereas brown adipocytes are rich in mitochondria. WAT is predominantly located in subcutaneous tissue, around internal organs, in the inguinal region, and the mesenteric area [15], where it stores excess metabolic energy as fatty acids [16]. BAT is mainly found in the neck, scapulae, and shoulder [17], and is responsible for reducing fat accumulation, increasing energy expenditure, and generating heat to aid weight loss by utilizing extra energy from metabolically active brown adipocytes. However, the amount of BAT in adults is minimal. Since WAT can transform into BAT under the action of browning agents, WAT has been regarded as a promising target for the treatment of obesity and complicated chronic diseases [18]. Overall, triggering the activation of BAT or inducing the browning of WAT offers a highly effective approach to obesity treatment [16,19].

The concept of using microneedles (MNs) for transdermal drug delivery was first patented by Gerstel and Place in 1976. Since the advent of advanced microfabrication technology in 1998, the first silicon MN array was produced using ion etching and was used for transdermal drug delivery [20]. An MN is a microarray of sharp needles supported by a base, capable of penetrating the epidermis and superficial dermis [21,22,23]. The resulting transient microchannels enable efficient drug delivery to the skin [24], overcoming the limitations of conventional transdermal preparations and alleviating the discomfort of subcutaneous injections. Over the past few decades, various MNs have been developed, mainly including four categories: drug-free solid MN [25], hollow MNs [26], drug-loaded solid MNs [27], and drug-loaded biodegradable MNs [28]. MNs are designed for transdermal drug delivery of various drugs, including small molecules [29], proteins [30], peptides [31], and nucleic acids [32]. Due to their minimally invasive nature, MN technology can avoid pain and tissue damage caused by injection, thereby improving patient compliance [33]. In addition, MNs avoid gastrointestinal adverse effects and hepatic first-pass metabolism involved in oral administration [34,35,36]. In recent years, increasing evidence has shown that the use of drug-loaded nanoparticles (NPs) can enhance drug bioavailability, as well as accumulation and retention of drugs in the targeted tissue, thereby improving therapeutic efficacy and reducing adverse effects [37,38,39,40,41]. Therefore, combining NPs with MN technology contributes to enhanced drug delivery efficiency.

**Figure 1:**
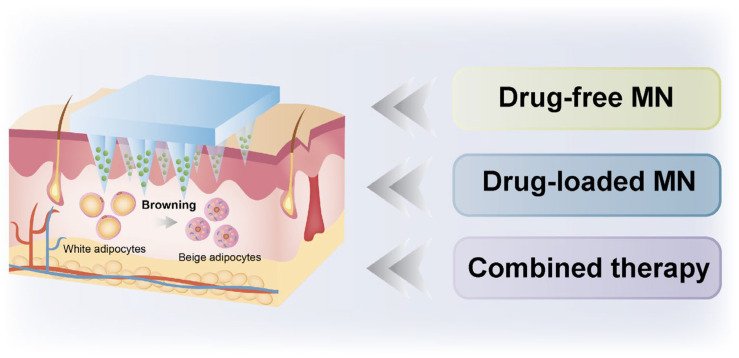
Schematic of various MN-mediated treatments for obesity.

In recent years, substantial advancements have been made in the field of MN mediated treatment of obesity, particularly in promoting the browning of adipose tissue, inhibiting adipogenesis, and improving metabolic functions. MNs penetrate the stratum corneum and precisely deliver drugs to the subcutaneous WAT to induce its browning, exerting effective action at the target site, significantly reducing systemic side effects and improving therapeutic outcomes. The administration of MNs is minimally invasive, painless, and safe, making MNs an ideal option for obesity treatment. Most MN drug delivery systems are passive, resulting in limited penetration depth of drugs into the dermal adipose tissue. Therefore, MN is often combined with other technologies to enhance drug delivery efficiency for obesity treatment, such as iontophoresis (INT) and photothermal therapy (PTT). INT is a non-invasive drug delivery technique that employs a low-intensity current to facilitate the entrance of charged molecules to the skin and enhance drug penetration depth, thereby improving drug delivery efficiency. PTT is another non-invasive treatment that converts light energy into localized heat, thereby inducing cell apoptosis. In the scenario of obesity treatment, PTT has been shown to promote the browning of white adipocytes, making it a promising therapeutic approach in this area. This review summarizes the latest progress of MNs in obesity treatment, including drug-free MNs and those that deliver drugs, NPs, etc., to promote the browning of WAT, inhibit adipogenesis, and improve metabolism. It also presents the integration of MNs with INT or PTT for combinatory anti-obesity treatment. Finally, the future development of MN technology is discussed (Figure 1).

## 2. Drug-Free MN for Obesity Treatment

Gelatin, as a natural polymer, has wide applications in drug delivery systems due to its good biocompatibility and biodegradability [42]. Although gelatin is widely used as a drug carrier or scaffold in the medical field, its use as a weight loss dietary supplement shows beneficial effects on fat metabolism. Gelatin is able to suppress lipogenesis in adipocytes while accelerating the process of lipolysis. Lipogenesis is a complex process in which preadipocytes differentiate into mature adipocytes. This process is regulated by the expression of key lipogenesis genes, including peroxisome proliferator-activated receptor gamma (PPARγ), CCAAT/enhancer-binding protein alpha (C/EBPα), and sterol regulatory element-binding protein-1c (SREBP-1c) [43]. Fatty acid synthase (FASN) is also referred to as a marker of terminal differentiation in adipocytes. The expression of FASN plays a critical role in lipogenesis, triggering the activation of PPARγ and SREBP-1c as part of a metabolic cascade [44].

**Figure 2:**
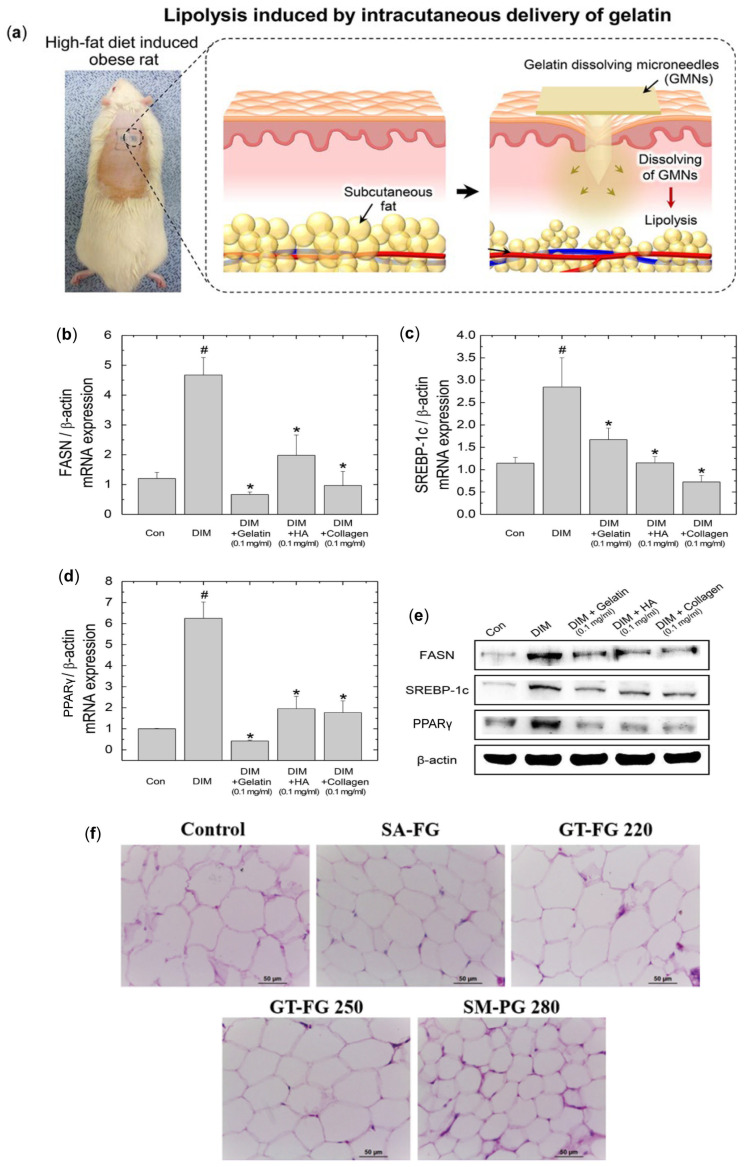
(**a**) Schematic illustration of a gelatin MN system for transdermal delivery aimed at reducing local subcutaneous fat. (**b**–**e**) Analysis of mRNA and protein expression levels of lipogenic genes in isolated rat adipocytes treated with three different polymers: gelatin, hyaluronic acid (HA), and collagen. Rat pre-adipocytes were differentiated into adipocytes using DIM media and subsequently exposed to polymers (0.1 mg/mL) for 24 h (* *p* < 0.05 compared to the DIM group; ^#^ *p* < 0.05 compared to the Con group). Transcription levels of lipogenic markers FASN (**b**), SREBP-1c (**c**), and PPARγ (**d**) were assessed by quantitative real-time qPCR and Western blotting (**e**) [45]. Reproduced with permission from [45], Copyright 2018, Acta Biomaterialia. (**f**) Hematoxylin and eosin (H&E) staining of subcutaneous adipose tissue treated with MN patches [46]. Reproduced with permission from [46] Copyright 2019, Toxicology Research.

Gelatin MN patches significantly improve the permeability of gelatin contents in the target area by forming microchannels in the skin. The locally released gelatin can modulate fat metabolism and effectively reduce fat accumulation. Although oral gelatin, as a dietary supplement, has been shown to be safe and effective in lowering plasma cholesterol and triglyceride levels in hypercholesterolemic rats, its metabolic pathways in the gut and liver are diverse, which limits its efficacy. In contrast, gelatin MN patches provide a more direct and effective treatment by locally acting on subcutaneous adipose tissue. Keum-Yong et al. [45] screened gelatin as a therapeutic polymer through in vitro experiments, isolating preadipocytes from the epididymal white adipose tissue (eWAT) of male Sprague-Dawley (SD) rats and inducing their differentiation into mature adipocytes in vitro (Figure 2a). The expression of adipogenesis-related genes, including *FASN*, *SREBP-1c*, and *PPARγ*, in adipocytes was assessed using real-time quantitative PCR (q-PCR) and western blotting (Figure 2b–e). The effect of gelatin on lipolysis was evaluated by a glycerol release experiment. The results showed that gelatin significantly decreased the expression of adipogenesis-related genes in adipocytes and increased glycerol release, indicating its promotion of lipolysis. Bullet-shaped gelatin MN (GMN) patches were also prepared and tested in an obesity rat model induced by a high-fat diet (HFD). The effects of GMN on the volume and morphology of subcutaneous WAT were evaluated using micro-computed tomography and histological analysis. The results showed that the application of GMN significantly reduced the subcutaneous WAT volume in obese rats, with local fat reduction up to 60%. Histological analysis revealed that the adipocyte volume decreased, and the cells became more contracted, indicating reduced fat deposition. Sung-Min et al. [46] used four types of gelatins: three derived from fish (SA-FG, GT-FG 220, and GT-FG 250) and one derived from pigs (SM-PG 280). They applied gelatin-based MN patches to a HFD-induced obesity rat model to evaluate their effects on subcutaneous fat reduction. The results showed that all four types of gelatin MN patches reduced the amount of subcutaneous WAT. Smaller adipocytes were observed in the areas treated with SA-FG, GT-FG 250, and SM-PG 280, indicating reduced fat accumulation (Figure 2f). Additionally, immunoblot analysis revealed that GT-FG 220 regulated FASN protein levels in subcutaneous adipose tissue, suggesting that gelatin MN patches reduce unwanted subcutaneous fat by altering lipid metabolism and fat deposition.

## 3. Drug-Loaded MN for Obesity Treatment

### 3.1. MNs Delivering Capsaicin for Obesity Treatment

Capsaicin (*trans*-8-methyl-*N*-vanillyl-6-nonenamide, Cap) is a primary bioactive compound responsible for the pungency of red peppers [47,48], showing multiple analgesia, anti-obesity, antibacterial, anti-inflammatory, and anticancer activities [49,50,51,52]. Recent studies have demonstrated that Cap can be used as a potential anti-obese medication to reduce weight by suppressing appetite and inducing satiety [53]. According to the findings of Krishnan et al. [54], the anti-obesity effects of Cap are mediated through the activation of transient receptor potential vanilloid 1 (TRPV1), upregulation of PPARγ expression, and a reduction in PPARγ acetylation levels, ultimately promoting the browning of white adipocytes. Furthermore, Joo et al. [55] demonstrated that Cap exhibits anti-obesity effects by enhancing the mRNA expression of uncoupling protein 1 (UCP1) in WAT and upregulating proteins associated with thermogenesis, lipid metabolism, and energy transduction. Taken together, Cap suppresses adipocyte differentiation [56], promotes the browning of white adipocytes [57], enhances thermogenesis, and decreases intracellular lipid levels [58], highlighting its potential as a promising anti-obese agent. However, the clinical application of Cap is hindered by its poor solubility and low oral bioavailability, which may result in adverse effects, including gastric ulcers, stomatitis, vomiting, and diarrhea [59]. Therefore, it is crucial to design a non-gastrointestinal drug delivery system targeting adipocytes.

**Figure 3:**
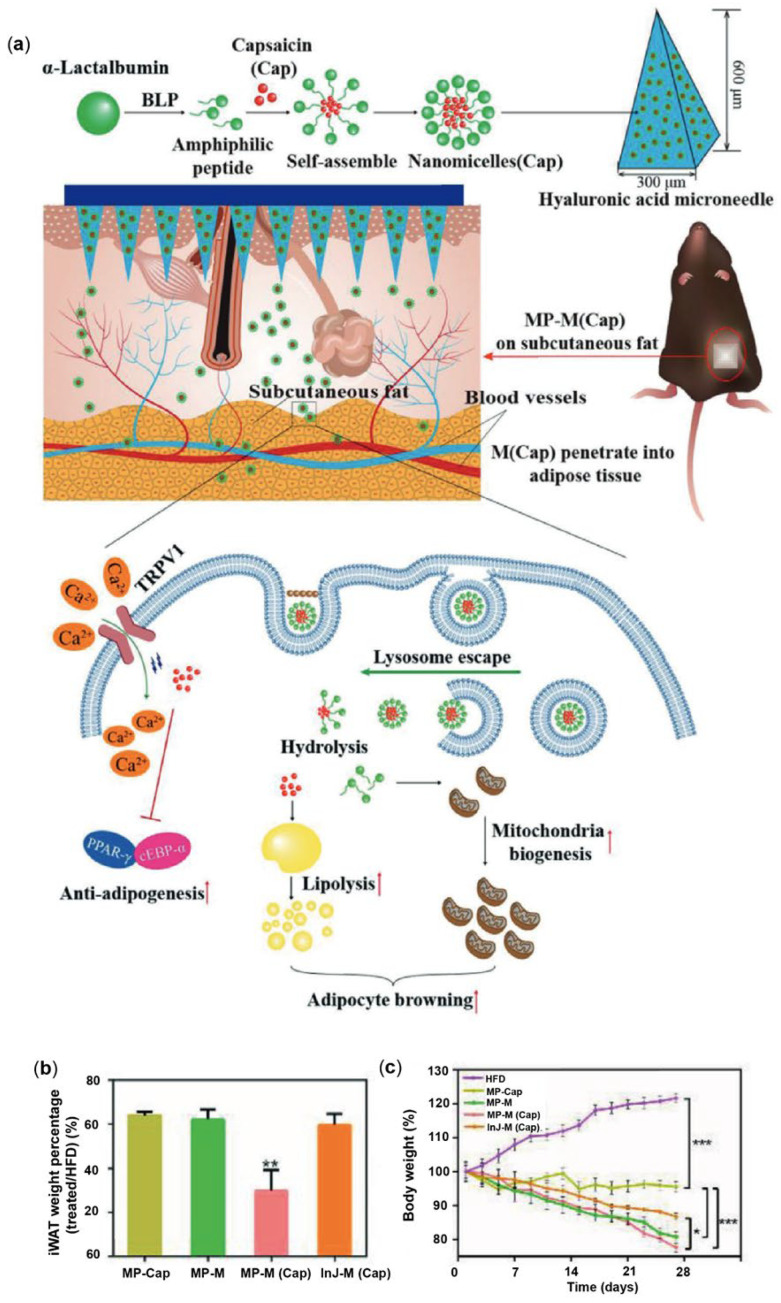
(**a**) Schematic representation of a multifunctional MN patch (MNP) incorporating Cap-loaded micelles designed to suppress adipogenesis and induce adipocyte browning. (**b**) Body weight progression of HFD-induced obese mice was monitored at specific time points following treatment with MP-M, MP-CAP, MP-M(CAP), and InJ-M(CAP) (** *p* < 0.01). (**c**) The relative weight of inguinal white adipose tissue (iWAT) in the treated groups was normalized to that of untreated HFD-induced obese mice (* *p* < 0.05, and *** *p* < 0.001) [60]. Reproduced with permission from [60] Copyright 2021, Advanced Functional Materials.

MN technology for transdermal drug delivery has been demonstrated as a minimally invasive and painless strategy for successful drug administration to adipose tissue. Bao et al. [60] developed and successfully applied a nanomedicine patch in which Cap was encapsulated in α-lactalbumin (α-lac) nanomicelles (M) and delivered directly to adipose tissue via an MN patch (Figure 3a). α-Lac materials are highly biocompatible and biodegradable [61], and their capacity to self-assemble into nanomicelles using readily available partially hydrolyzed peptides, while encapsulating significant amounts of hydrophobic compounds, makes them particularly appealing for nanomedicine applications [62]. The experiments also revealed the ability of nanomicelles to evade lysosomal degradation and that α-lac micelles themselves can inhibit PPARγ and C/EBPα activity, facilitating the accumulation of Cap receptors while increasing levels of transient receptor potential TRPV1, UCP-1, and cytochrome C. The inhibitory effects of Cap-encapsulated nanomicelles (M (Cap)) on adipogenesis and their browning promotion capability were assessed using the 3T3-L1 adipocyte model. Findings indicated that M (Cap) reduced lipid droplet accumulation through the modulation of adipogenesis and improved mitochondrial biogenesis. Additionally, the MN patch facilitates the efficient penetration of M (Cap) into subcutaneous abdominal adipose tissue, where M (Cap) can be readily endocytosed by white adipocytes. The overall anti-obesity effect of the MN delivery system was evaluated using a HFD-induced obese mouse model. After 28 days of treatment, all treated groups exhibited a partial reduction in body weight compared to the untreated obese mice (Figure 3b). In particular, mice treated with the MN patch delivering encapsulated Cap nanomicelles (MP-M(Cap)) showed the most significant weight loss (Figure 3c). Magnetic resonance imaging (MRI) analysis revealed that the MP-M(Cap) treatment group had the lowest body fat content, indicating that this system not only reduced overall body weight but also decreased body fat. MP-M(Cap) treatment has been shown to activate energy metabolism, enhance mitochondrial biogenesis, and upregulate established markers of adipocyte browning. These results highlight MP-M(Cap) as a promising non-invasive therapeutic strategy for obesity management [60].

### 3.2. MNs Delivering Botulinum Toxin for Obesity Treatment

In recent years, neuroregulation has become a minimally invasive strategy for inhibiting gastrointestinal motility. Botulinum Toxin Type A (BTX-A) is an effective muscle contraction inhibitor, and its role in obesity treatment mainly involves the following aspects: (1) Inhibition of gastrointestinal motility: BTX-A inhibits gastrointestinal motility by interfering with the release of acetylcholine (Ach) [63]. The compound binds to the botulinum toxin receptor and is subsequently endocytosed into cholinergic nerve terminals [64]. Within the terminals, it cleaves the soluble *N*-ethylmaleimide-sensitive factor attachment protein receptor (SNARE) complex, thereby inhibiting the exocytosis of Ach-loaded synaptic vesicles. This action suppresses excitatory postsynaptic potentials, resulting in the localized inactivation of effector cells, including smooth muscle and glandular cells [65]. Neurogenic gastrointestinal events primarily depend on the excitatory neurotransmitter Ach, thus inhibiting gastrointestinal motility; (2) Reduction in gastric emptying rate: Ach deficiency induced by BTX-A significantly reduces gastric motility in the stomach, including phase contractions and the resulting peristaltic waves, both of which are related to the gastric emptying rate [66,67]. The decrease in gastric emptying rate can reduce food digestion and absorption, thus influencing body weight; (3) Metabolic improvement: BTX-A can improve hepatic fat degeneration, reduce blood lipids, and enhance gut microbiota, leading to positive effects on obesity-related metabolic disorders. The effects of BTX-A are specific, reversible, and do not produce significant side effects.

**Figure 4:**
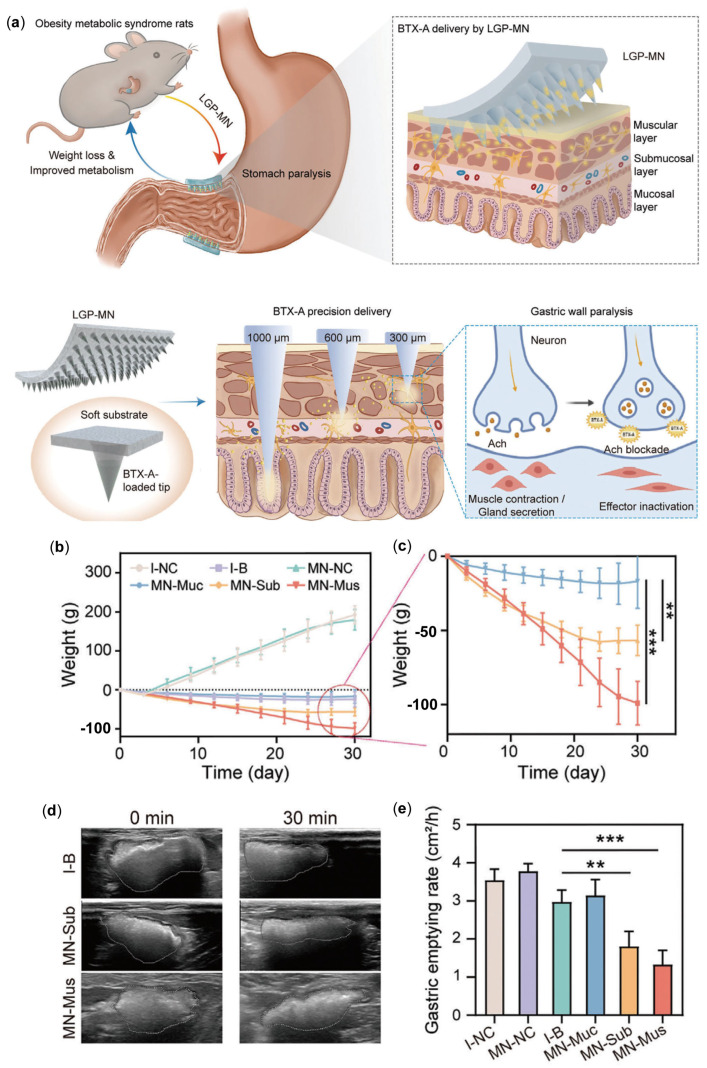
(**a**) Conceptual illustration of the application and mechanism of LGP-MN. (**b**,**c**) Detailed schematic explanation of the mechanism and application of LGP-MN (*n* = 7–8, ** *p* < 0.01, and *** *p* < 0.001). (**d**) Ultrasound images showing gastric volume changes two weeks post-BTX-A treatment. (**e**) Statistical analysis of gastric emptying rates measured via ultrasound [69]. Reproduced with permission from [69] Copyright 2023, Advanced Science.

It has been reported that local injection of BTX-A reduces gastric wall muscle contraction, thereby slowing gastric emptying. However, the weight loss effect induced by BTX-A injection varies greatly under different conditions [68], leading to pain at the injection site and possible allergic reactions. Given that the gastric wall consists of three distinct layers—muscular, submucosal, and mucosal—each with unique structures and functions, it is possible that they may exhibit varying responses to BTX-A. The inconsistent therapeutic effect of BTX-A may stem from variations in injection depth and uneven drug distribution within the gastric wall. To fully harness the weight loss effects induced by BTX-A following gastric administration, it is essential to develop uniform and precise delivery strategies that allow for targeted study of its neuroinhibitory effects across each gastric layer. Wang et al. [69] developed layer-specific gastric paralysis MNs (LGP-MNs) for the precise delivery of BTX-A to three different gastric wall layers. The drug-loaded tips of LGP-MNs quickly release BTX-A, ensuring uniform distribution within the targeted gastric wall layers and achieving high BTX-A concentrations locally (Figure 4a). Utilizing the layer-specific delivery strategy, the differential therapeutic effects of BTX-A administered to the three gastric wall layers were demonstrated: (1) The most direct effect of BTX-A delivered to the mucosal layer is the inhibition of presynaptic nerve terminal release of Ach and the loss of excitatory postsynaptic potentials; (2) BTX-A delivered to the submucosal layer inhibits gastric emptying by blocking Ach release and suppressing glandular cells; (3) BTX-A delivered to the muscular layer directly causes muscle paralysis, thereby reducing gastric motility. In an obesity rat model, LGP-MNs not only proved safer than conventional injection methods but also exhibited sustained and superior therapeutic outcomes in muscle layer delivery, including a 16.23% weight loss (3.06 times greater than conventional injections) (Figure 4b,c), a 55.20% reduction in gastric emptying rate, improved hepatic fat degeneration, and reduced blood lipid levels (Figure 4d,e). Additionally, it was found that muscle layer-targeted LGP-MN treatment improves glucose tolerance by increasing the production of gastric-derived glucagon-like peptide-1 (GLP-1), offering unique effects. This finding provides a new theoretical basis for the application of BTX-A in weight management and metabolic improvement.

### 3.3. MN Delivering Rosiglitazone for Obesity Treatment

Rosiglitazone (Rosi) is a type of thiazolidinedione (TZD)medication that is commonly used as an insulin sensitizer for the treatment of diabetes. Rosi can activate the intracellular receptor of PPARγ, which plays a crucial role in modulating glucose and lipid homeostasis, enhancing insulin responsiveness, and reducing inflammation [70,71]. In obesity, subcutaneous WAT contains large unilocular lipid droplets that store excess energy as triglycerides. During obesity treatment, PPARγ agonists can convert white adipocytes into brown adipocytes [72]. The weight-reducing capacity of brown adipocytes is attributed to their unique cellular characteristics. These cells are rich in mitochondria, exhibit robust metabolic activity, and express elevated levels of UCP1, collectively facilitating efficient energy expenditure and thermogenesis, which helps inhibit mitochondrial oxidative phosphorylation and enhances the catabolism of free fatty acids [73]. Within adipose tissue, ligand binding to PPARγ orchestrates the differentiation of preadipocytes into mature adipocytes. A hallmark effect of TZD treatment is the expansion of small, insulin-sensitive adipocytes, as reported in several studies [74,75]. Rosi has been shown to augment insulin-stimulated glucose transport by upregulating the expression of glucose transporter 4 (GLUT4) and modulating the expression of cytoskeletal proteins that facilitate the fusion of GLUT4-containing vesicles with the plasma membrane. However, various agents that promote “browning” may have broad targeting spectra and cause adverse effects on other organs. Rosi has significant side effects, with some irritation to the gastrointestinal tract, potentially exacerbating gastrointestinal reactions. Local delivery of the drug can minimize its adverse effects, but due to off-target effects, the effectiveness of Rosi is quite limited.

**Figure 5:**
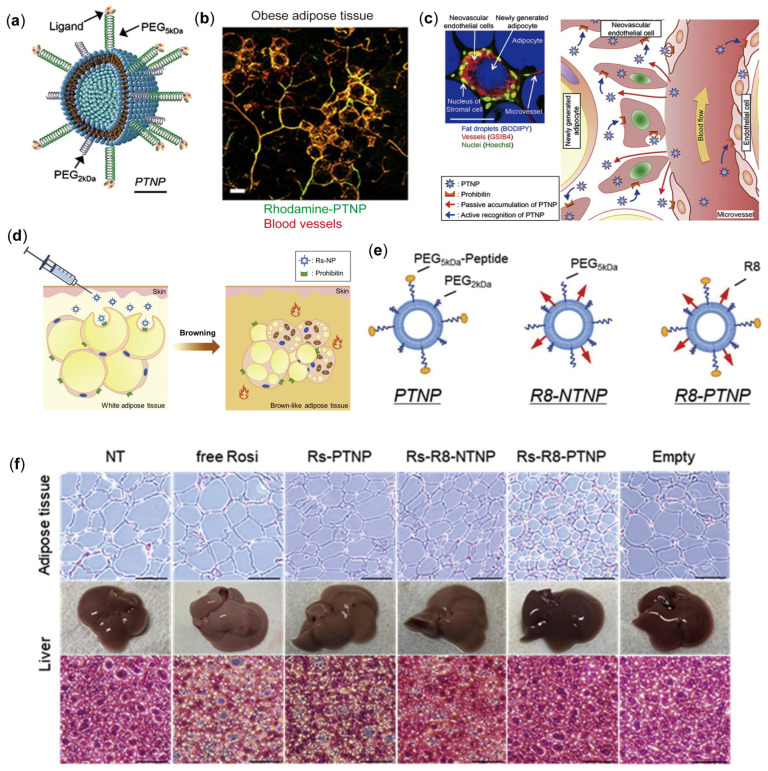
(**a**) Schematic representation of the design of vascular-targeted NPs (PTNP: prohibitin-targeted NPs). (**b**) Illustration of the enhanced targeting of PTNP to white fat vasculature (WFV) in diet-induced obese (DIO) mice through active and passive mechanisms (scale bars: 100 μm). (**c**) Diagram of the dual mechanisms—active and passive targeting—enabling improved drug delivery of PTNP into obese adipose tissue [79]. Reproduced with permission from [79] Copyright 2012, Journal of Controlled Release. (**d**) Schematic depiction of dual-targeted Rs-NPs promoting the browning of WAT. (**e**) Conceptual design of three types of NPs. (**f**) Histological analysis of iWAT (upper panel, scale bars: 100 μm) and liver tissue (lower panel, scale bars: 50 μm) stained with H&E following administration of free Rosi and various Rs-NPs; representative images of excised livers are presented at the conclusion of the treatment period [84]. Reproduced with permission from [84] Copyright 2021, Journal of Controlled Release.

NPs serve as versatile drug carriers that can be functionalized with targeting components to direct drug delivery to specific tissues or cell types, thereby enhancing drug accumulation at desired sites [76,77]. Consequently, nanoparticles (NPs) emerge as a powerful platform for targeted drug delivery. Through phage display screening, the peptide sequence CKGGRAKDC was identified as a ligand for an inhibitory protein that is highly expressed in the endothelial cells of WAT [78]. Hossen et al. [79] developed inhibin-targeting NPs (PTNP), a liposomal platform functionalized with inhibin-binding peptides, which can facilitate the selective delivery of therapeutic agents to endothelial cells within WAT by specifically binding to inhibin, thereby enhancing the precision and efficacy of drug targeting [80,81]. Recent studies have indicated that the inhibitory protein is expressed not only in the blood vessels of WAT but also in mature white adipocytes (Figure 5a–c) [82,83].

Hiradate et al. [84] developed dual-targeted Rosi-loaded NPs (Rs-NPs) that specifically target adipocytes (Figure 5d). These NPs enhance cellular uptake and control intracellular transport by combining inhibin-binding peptides and cell-penetrating peptides (such as octa-arginine, R8). Three types of Rs-NPs were prepared, including inhibin-targeted NPs (PTNP), non-targeted NPs with cell-penetrating peptides (R8-NTNP), and dual-targeted NPs (R8-PTNP) (Figure 5e). Rosi was encapsulated into NPs via the reverse evaporation method, and its uptake and browning ability in 3T3-L1 cells were evaluated. The experimental results showed that R8-PTNP exhibited significant uptake in mature adipocytes (Figure 5f). To develop a new therapy for the management of obesity through enhancement of energy expenditure, it is crucial to induce the browning of WAT while minimizing the adverse effects of Rosi on other organs, such as the liver and kidneys. Systemic administration of Rs-NPs may prove insufficient for delivering the drug directly to adipocytes within WAT. This limitation arises because a significant proportion of intravenously administered Rs-NPs tend to accumulate in the endothelial cells lining WAT blood vessels, thereby impeding efficient drug delivery to the target adipocytes [79]. Moreover, R8-modified liposomes are prone to hepatic accumulation, primarily due to recognition by opsonin or macrophages [85]. To mitigate the off-target effects of Rosi while effectively promoting the browning of WAT, it is essential to ensure that the majority of encapsulated Rosi reaches adipocytes. Local administration of Rs-NPs directly into WAT is anticipated to deliver a minimal yet sufficient concentration of Rosi to adipocytes. This approach is expected to facilitate robust beige fat biogenesis, thereby treating obesity without eliciting adverse effects.

**Figure 6:**
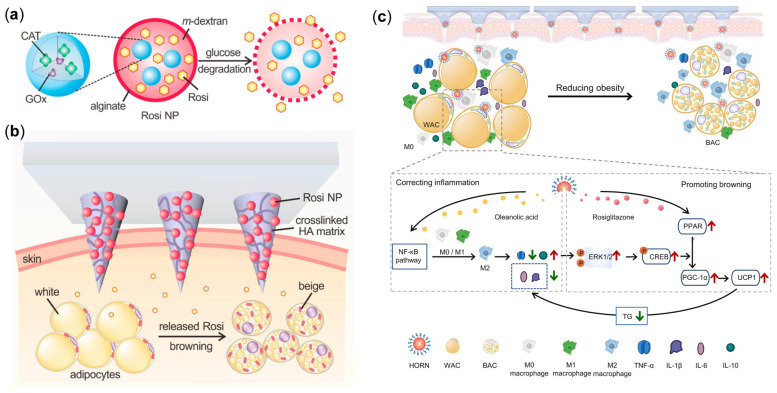
(**a**,**b**) Schematic illustration of an MN patch integrated with browning reagents. NPs encapsulating Rosi, GOx, and CAT are synthesized using pH-sensitive dextran functionalized with acetal linkages and encapsulated with alginate. These NPs are incorporated into a MN-array patch composed of a HA matrix stabilized through cross-linking for inducing browning in WAT [18]. Reproduced with permission from [18] Copyright 2017, ACS Nano. (**c**) Mechanistic diagram of the soluble nanoparticle MN patch for obesity treatment [86]. Reproduced with permission from [86] Copyright 2024, Biomaterials.

The MN patch-based local induction of fat tissue browning technology can reduce obesity and its complications through localized drug delivery. Zhang et al. [18] developed an MN patch capable of successfully delivering glucan NPs containing the potent insulin sensitizer compound Rosi, enabling local treatment of iWAT and resolving the toxicity problems that hinder the clinical implementation of Rosi. Degradable NPs were fabricated utilizing acid-sensitive dextran derivatives via a double emulsion process, with Rosi incorporated as a browning agent. These NPs were then embedded within the MN array to achieve sustained drug delivery to subcutaneous adipose tissue (Figure 6a,b). Additionally, glucose oxidase (GOx) and catalase (CAT) were employed to generate an acidic microenvironment under physiological glucose concentrations, which triggers the release of encapsulated drugs. In in vitro experiments, it was confirmed that Rosi-loaded NPs, similar to unencapsulated Rosi, effectively induced the formation and browning of adipocytes. In a pharmacodynamic study using a HFD-induced obesity mouse model, the MN patch significantly reduced the size of fat pads, increased overall energy expenditure, and improved metabolism. Moreover, the application of Rosi MN and CL 316243 MN (another β3-adrenergic receptor agonist) in mice resulted in reduced adipocyte magnitude and increased energy consumption, leading to weight loss and improved insulin sensitivity. Chen et al. [86] prepared Rosi NP dissolving MN patches (HORN-MN) with weight-loss function using synthetic oleanolic acid dimers as NP materials. HORN-MN can effectively penetrate the skin’s stratum corneum, dissolve within the abdominal dermis in under 5 min, and release Rosi NPs. Subsequently, the NPs are internalized by macrophages and WAT, and then degrade into oleanolic acid sequestered within lysosomal compartments, releasing Rosi. Oleanolic acid significantly improves the inflammatory state of adipose tissue and promotes the browning of WAT, while Rosi significantly enhances this process (Figure 6c). In the mouse model, HORN-MN substantially decreased body weight and total adiposity, improving serum biochemical indicators associated with obesity, including lowering plasma glucose, cholesterol profile, serum triglycerides, and low-density lipoprotein cholesterol levels, while increasing high-density lipoprotein levels. Histological analysis revealed that mice treated with HORN-MN exhibited increased UCP1 protein expression in iWAT, along with reduced adipocyte size, indicating successful browning of white adipocytes. These findings suggest that MN patch delivery of Rosi is an effective obesity treatment method that improves metabolic health by promoting the browning of WAT, offering a novel therapeutic avenue for the clinical management of obesity and its associated metabolic disorders.

### 3.4. MN Delivering Liraglutide for Obesity Treatment

Liraglutide (LRT) is a GLP-1 receptor agonist, which is not only effective in lowering glucose but also has the effects of weight loss, lowering blood pressure, and improving the lipid profile. Its clinical application is becoming more widespread, and it has been endorsed for weight reduction in the management of obesity [87]. GLP-1 is a type of intestinal pro-insulin endogenously present within the human body: it exerts a role in facilitating weight reduction by agonizing widely distributed GLP-1 receptors, but it is extremely easy to degrade. LRT can enhance insulin secretion by binding to GLP-1 receptors and inhibit glucagon secretion to promote glucose consumption and reduce energy intake. LRT encourages weight loss by binding to the GLP-1 receptor, enhancing insulin secretion, inhibiting glucagon secretion, and reducing calories by promoting glucose consumption; suppressing appetite through the nerve center to reduce eating; delaying gastric emptying and gastrointestinal peristalsis; and prolonging the time to satiety. In clinical applications, LRT is usually administered by subcutaneous injection, which has a relatively good safety profile; however, patients can experience gastrointestinal reactions such as nausea, vomiting, and diarrhea, and long-term injections can result in significant biohazardous waste, as well as an elevated likelihood of complications, including lipoatrophy, inflammation, and the formation of subcutaneous nodules, and infections [88]. In addition, the correlated poor adherence typically leads to suboptimal therapeutic efficacy. Therefore, there is a need to develop a new dosage form for the delivery of LRT for the treatment of obesity.

**Figure 7:**
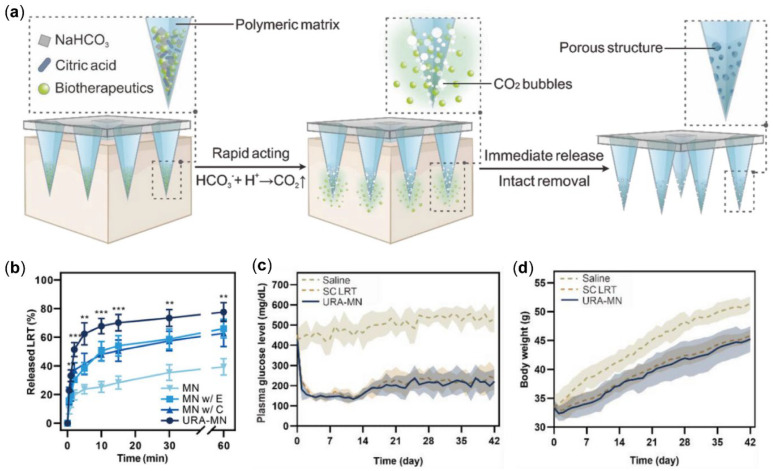
(**a**) Schematic representation of the ultrarapid-acting MN (URA-MN) patch, engineered to facilitate the immediate delivery of biotherapeutics. (**b**) Dissolution kinetics of LRT from diverse MN patches, with each group comprising three replicates (*n* = 3, ** *p* < 0.01, and *** *p* < 0.001). (**c**) Plasma glucose levels in db/db mice (*n* = 5) treated daily with saline, subcutaneous LRT injection (1.00 mg/kg), or MN patches (1.73 mg/kg) over six weeks. (**d**) Body weight changes in db/db mice (*n* = 5) monitored over the same period [92]. Reproduced with permission from [92] Copyright 2023, Advanced Materials.

MN has garnered significant attention as an efficacious and minimally invasive platform for the transdermal delivery of therapeutic agents, enabling efficient transport across the skin barrier while mitigating the risk of substantial dermal trauma [89,90,91]. You et al. [92] developed ultra-fast-release pneumatic MN for LRT delivery, which was developed by adding a vesicant to the MN matrix and distributing the vesicant and drug at the tip of the needle through centrifugal force (Figure 7a). When the MN comes into contact with skin tissue fluid, the vesicant absorbs water and generates a large amount of gas, which accelerates the release and transdermal diffusion of LRT, thus helping patients to complete the administration of the drug in a few minutes and improving medication adherence. In vitro release results showed that the pneumatic MN significantly accelerated the release of LRT compared to the other non-pneumatic MN groups, with more than 60% of the drug being released within 10 min (Figure 7b). In addition, the researchers elucidated in a murine model of type 2 diabetes that the MN is biocompatible and achieves therapeutic efficacy comparable to that of subcutaneous injection. After 6 weeks of continuous use, the mice showed a 33% inhibition of body weight gain and an approximately 58% reduction in blood glucose compared to the control group (Figure 7c,d). The developed ultra-fast-release pneumatic MN showed higher biocompatibility and fewer adverse reactions, including local fibrosis and inflammation, in extended administration compared to conventional daily subcutaneous injections.

## 4. Combination Therapy

### 4.1. Microneedling Combined with INT for Obesity Treatment

Iontophoresis (INT) is a non-invasive drug delivery modality that employs a low-intensity electric current to facilitate the transdermal transport of charged molecules. This technique typically involves the application of a current ranging from 0.1 to 1.0 mA/cm^2^, which generates a potential gradient that propels ionic drugs across the skin through electrostatic interactions. By leveraging an electric current, INT enhances the penetration of charged therapeutic agents through the skin barrier, thereby offering a viable alternative to conventional needle-based injectable delivery methods [93]. Ion electroosmotic transport encompasses two main mechanisms: electrokinetic migration (also known as electro-rejection) and electroosmosis. Electrokinetic migration involves the movement of ions towards an oppositely charged electrode. In contrast, electroosmosis involves the migration of water molecules from the positively charged anode to the negatively charged cathode [94]. Drug concentration, overall charge, molecular physicochemical attributes, molecular volume, solubility partition coefficient, and area of skin employed for application all affect iontophoretic drug delivery, and the effectiveness of iontophoretic transfer is also influenced by skin surface intactness, stratum thickness, and area-specific differences in blood flow in different areas of the skin [94,95]. INT is usually associated with mild adverse effects instead of serious adverse effects. When appropriate application strategies are utilized, the treatment is usually well sustained [96,97].

One of the most important developments in transdermal therapeutic systems involves the synergistic integration of multiple enhancement technologies to achieve a cumulative effect [98,99]; for example, combining MN with INT has shown significant potential in improving the efficiency of transdermal therapeutic delivery [100]. MN generates low-resistance water channels in the skin, which can be further utilized by INT to increase the flux of drugs through the skin layer [98,101]. Previous investigations have demonstrated that the synergistic integration of MN and INT increases the penetration of drugs delivered transdermally compared to a singular strategy [102,103,104,105,106]. The efficacy of these integrated strategies is especially significant for the delivery of large molecules, which have traditionally faced considerable obstacles in transdermal delivery limited by their size. INT can also be used to better enhance regulation of drug flux by modulating the imposed current, reducing the delay associated with drug penetration, and providing adaptability in facilitating the delivery of an extensive array of therapeutic agents, encompassing small molecules, macromolecules, and NPs [101,107,108]. More sophisticated and user-friendly wearable INT-driven MN patches for transdermal drug delivery have recently been innovatively developed, integrating the physical penetration capability of MN with electrically-mediated iontophoretic drug delivery within a singular wearable apparatus [105,109,110]. These amalgamated systems enable precise electrically controlled drug delivery in a user-friendly wearable format, which has the potential to improve therapeutic outcomes, especially in chronic diseases. A study by Singh et al. [111] found that INT-assisted MN delivery substantially improved the percutaneous delivery of ropinirole hydrochloride. This method provided precise control of dose titration in the treatment of Parkinson’s disease. In addition, Junaid et al. [112] have elucidated the utility of INT-assisted MN for baclofen delivery in the management of multiple sclerosis. This transdermal modality offers a painless and convenient means of drug administration, thereby enhancing patient adherence and safety while achieving therapeutic baclofen concentrations. Similarly, this approach markedly augments the transdermal permeation and systemic absorption of NSAIDs, including diclofenac sodium [113] and ibuprofen sodium [114]. This strategy, especially when combined with hydrogel-forming MN, enhances the delivery of these analgesic and anti-inflammatory agents, potentially providing a more effective therapeutic option. Another investigation by Arshad et al. [115] illustrated that the combination of MN-mediated delivery of dexamethasone sodium phosphate, augmented by INT, significantly attenuated edema in the hind paw of rats and was more effective in decreasing pro-inflammatory cytokines, as compared to the use of MN alone. The combination of MN and electrical methods showed synergistic improvement in vivo, effectively facilitating the delivery of hydrophilic macromolecules while preserving skin integrity.

**Figure 8:**
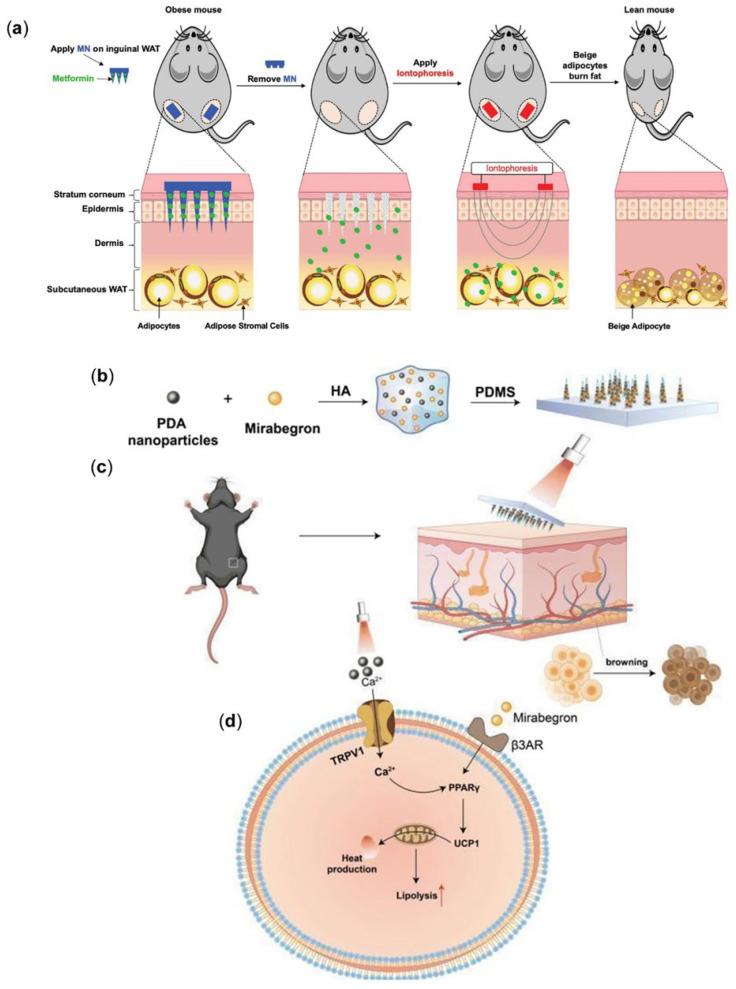
(**a**) Schematic representation of metformin delivery to subcutaneous WAT in obese C57BL/6J mice using a soluble PLGA MN assisted by INT to promote WAT browning and thermogenesis [104]. Reproduced with permission from [104]. Copyright 2022, Pharmaceutics. (**b**–**d**) Illustration of transdermal photothermal drug therapy using MN patches loaded with polydopamine NPs (PDA-NPs) and mirabegron to suppress adipogenesis and induce adipocyte browning. (**b**) Preparation process of the MN patch. (**c**) Step-by-step procedure for anti-obesity MN treatment. (**d**) Mechanism underlying the browning of WAT induced by the MN patch therapy [121]. Reproduced with permission from [121]. Copyright 2024, Biomaterials Science.

In obesity treatment, INT is often utilized in combination technology to improve drug administration efficiency and targeting. When the drug is administered transdermally via MN, the drug belongs to passive diffusion and penetration; then, the penetration depth of the drug in the skin tissue is limited, and the drug delivery efficiency can be improved by increasing the penetration depth of the drug in the dermis layer of the adipose tissue through INT technology. INT enhances the transdermal delivery of polar hydrophilic therapeutic molecules by 10- to 2000-fold compared to conventional topical application. It has been proposed that the synergistic combination of MN and INT significantly increases the number of therapeutic molecules delivered by dissolving MN patches. Consequently, the delivery efficiency of this integrated approach is markedly superior to that of either modality used in isolation. In a study by Abbasi et al. [104], metformin was successfully delivered directly into the subcutaneous WAT of obese mice by using dissolvable polylactic acid-hydroxyacetic acid copolymer (PLGA) MN and INT (Figure 8a). Metformin is known as an AMPK activator that combats obesity by promoting WAT browning and enhancing metabolic activity. The study found that the co-administration of these two approaches was more impactful in reducing body weight and fat accumulation, augmenting energy utilization, and improving energy metabolism in mice than microneedling or INT alone. By using this MN + INT approach to browning of subcutaneous WAT via the targeted delivery of metformin and additional browning agents, it may be possible to mitigate obesity with an effective, simple, and safe protocol.

### 4.2. Microneedling Combined with PTT for Obesity Treatment

PTT, a non-invasive therapeutic technique that converts light energy into localized heat to induce apoptosis, has garnered increasing attention due to its precise and controllable treatment sites, high killing efficiency, and minimal side effects [116,117] and holds promising potential for applications in the treatment of malignant tumors and infectious diseases [118]. Recent investigations have demonstrated that mild thermal stimulation of adipose tissue can effectively activate the transient receptor potential TRPV1 channel, thereby promoting Ca^2+^ influx, which in turn activates PPARγ and increases the expression of UCP 1, thereby promoting browning of WAT, suggesting that PTT has great potential in the treatment of obesity. For example, Zhang et al. [119] proposed a localized PTT in combination with cationic albumin NPs with adipocyte affinity for the targeted delivery of Rosi to WAT, thereby inducing the browning process and thus treating obesity. Thermosensitive hydrogels loaded with IR 780 were injected and gelled in situ to provide subcutaneous reservoirs, which enabled local PTT and controlled release of RSG cNP. In vitro studies showed that cationic NPs improved the efficiency of adipocyte internalization, while in vivo studies showed that they prolonged retention in adipose tissue. In diet-induced obese mice, hydrogels loaded with RSG cNPs and subjected to local PTT effectively reduced adiposity and induced browning of WAT, while attenuating complications such as insulin resistance, fatty liver, and hyperlipidemia. This therapeutic strategy, which integrates topical PTT with targeted delivery to adipocytes, offers a promising solution to combat the global obesity epidemic. By minimizing systemic toxicity and enhancing therapeutic efficacy, this approach holds significant potential for clinical translation.

The current MN patch technology combined with PTT offers an innovative solution for the treatment of obesity by inducing fat browning to achieve weight loss. Among a diverse array of photothermal materials, polydopamine nanoparticles (PDA-NPs) exhibit superior biodegradability, biocompatibility, photothermal stability, and high photothermal conversion efficiency [16,120]. As an excellent photothermal conversion material, when exposed to near-infrared (NIR) laser irradiation, the PDA-NPs convert light energy into geothermal energy, thereby activating TRPV1 channels. This patch acts directly on subcutaneous WAT and induces the browning of WAT through mild PTT. In a recent investigation, Gao et al. [121] developed an MN patch loaded with polydopamine NPs and migration (Figure 8b–d). It was shown that this method not only promoted fat browning but also had good biocompatibility. In the experiment, untreated mice gained 9% of body weight, while obese mice treated with PTT lost nearly 19% of body weight. When mirabilone was used in combination with PTT, the weight loss in the obese mice was even more dramatic, reaching a weight loss of about 22 percent.

## 5. Conclusions and Prospects

Obesity has become a global public health problem, while there are still no effective treatments that can prevent weight rebound. MN has demonstrated substantial potential in the realm of obesity management. This review summarizes the latest progress in the field of MN-mediated obesity treatments, including drug-free MN and the use of MN to deliver drugs, NPs, etc., to promote browning of WAT, inhibit adipogenesis, and improve metabolism. MN technology that precisely targets adipose tissue significantly enhances anti-obesity effects while reducing systemic side effects. With the development of nanotechnology that enables targeted drug delivery, controlled drug release, and enhanced drug bioavailability, the integration of MN with nanotechnology has attracted increasing attention in managing obesity.

Although MN technology has made significant progress in obesity treatment, future research needs to further explore the safety, efficacy, and optimal dosing regimen of MN technology in order to achieve satisfying anti-obesity effects. In addition, combination therapy that integrates MN technology and INT or PTT provides alternative options for obesity treatment with enhanced therapeutic efficacy. With the continuous breakthrough of nanotechnology and biomaterials, MN technology is expected to become an important means of obesity treatment and provide more personalized treatment options for patients.
